# Massive Hemothorax Following Transradial Coronary Angioplasty

**DOI:** 10.7759/cureus.80236

**Published:** 2025-03-07

**Authors:** Ankit Jain, Aakash Vijay, Anil Yadav, Devesh Kumar, Anwar Ansari

**Affiliations:** 1 Cardiology, Vardhman Mahavir Medical College and Safdarjung Hospital, Delhi, IND

**Keywords:** bleeding complication, coronary artery angiography, hydrophilic wire, massive hemothorax, trans-radial pci

## Abstract

Hemothorax is a rare complication following coronary angioplasty. We report a case of a young adult who presented with anterior wall myocardial infarction, underwent coronary angioplasty, and subsequently developed massive right-sided hemothorax. It was suspected clinically and a chest radiograph and contrast-enhanced computer tomography (CECT) of the thorax confirmed the diagnosis. Injury to the right subclavian and right internal mammary arteries was subsequently ruled out by selective angiography. Despite our best efforts, we could not find any evidence of perforation or any bleeding site; therefore, the patient was successfully managed conservatively with intercostal drainage (ICD) and blood transfusion. We review this rare complication and its differentials and briefly discuss the literature.

## Introduction

Massive hemothorax, though a rare complication, can occur after transradial angioplasty, an access site that is now preferred for coronary angioplasty. Radial access is preferred over femoral access due to its reduced complication rates and faster recovery. Hemothorax, defined as the accumulation of blood in the pleural cavity, typically results from iatrogenic injury. In the context of transradial angioplasty, it arises due to vascular injuries, such as perforation of the subclavian artery or internal mammary artery during catheter manipulation, or from the rupture of small collateral vessels [1].

The incidence of hemothorax following transradial procedures is extremely low, yet it carries significant morbidity due to the potential for rapid blood loss and respiratory compromise. Clinically, patients may present with sudden-onset chest pain, dyspnea, and signs of hemodynamic instability. Radiologically, a hemothorax is diagnosed using a chest radiograph or computed tomography of the chest, which reveals a large fluid collection in the pleural space [2].

Management of massive hemothorax requires prompt recognition and intervention. Herein, we report a case of massive hemothorax during transradial coronary angioplasty and subsequently its successful management. Moreover, we briefly review transradial access-related perforations and other complications.

## Case presentation

**Figure 1:**
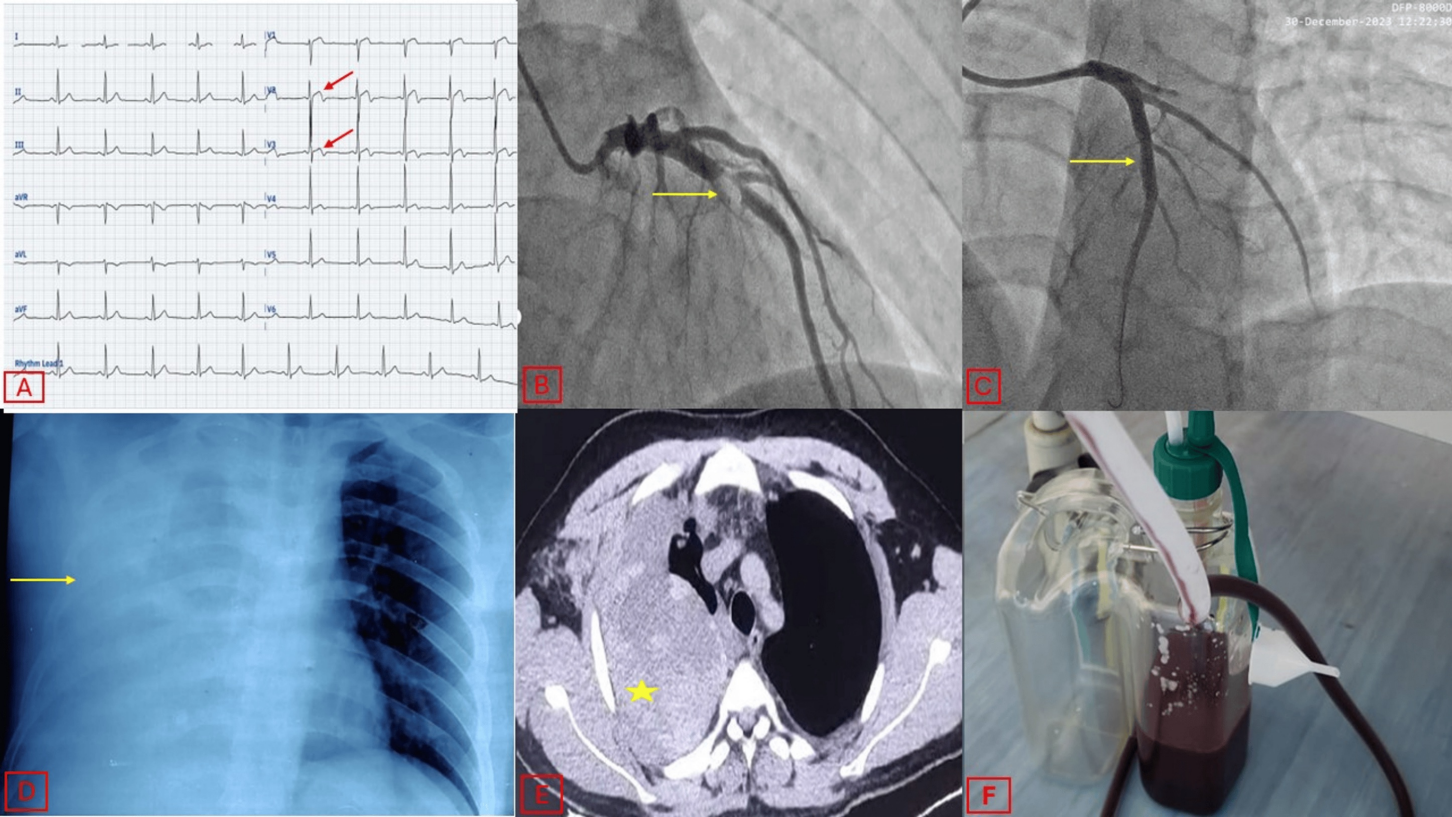
Summary of the case. A) 12-lead electrocardiogram showing biphasic T waves in leads V1-V3, consistent with Type A Wellen's pattern (red arrows). B) Coronary angiogram of the left coronary system in the right anterior oblique cranial view using a 5 French Tiger catheter, showing a Grade IV thrombus (yellow arrow) in the mid-segment of the left anterior descending artery. C) Coronary angiogram of the left coronary system in the right anterior oblique cranial view using a 6 French Judkins left (JL3) catheter, post-deployment of a stent (yellow arrow), with TIMI 3 flow distally. D) Chest radiograph in the PA view showing right hemithorax completely white-out (yellow arrow). E) Transverse section of chest computed tomography images showing a collection in the right hemithorax with attenuation of 35-70 HU (yellow star), associated with underlying passive lung collapse. F) 500 mL of blood drained out post-ICD insertion in the right-sided hemothorax. PA, posteroanterior; ICD, implantable cardioverter-defibrillator; TIMI, thrombolysis in myocardial infarction

A young male in his 20s presented to the emergency department with complaints of left-sided chest pain radiating to the left shoulder for the past 5 hours. At the time of presentation, the patient was hemodynamically stable. His cardiovascular examination and other systemic examinations were normal. Upon further investigation, his 12-lead electrocardiogram revealed a type A Wellen’s pattern with biphasic T waves in leads V2-V3 (Figure 1A). 2D transthoracic echocardiography showed mild left ventricular dysfunction with regional wall motion abnormalities in the anterior, anteroseptal, and anterolateral walls at the mid and apical levels. Serum troponin levels were elevated to a value 10 times the upper limit of normal.

The patient was taken to the catheterization laboratory for percutaneous coronary intervention via 6 French radial artery access. Diagnostic coronary angiography was performed using a 5 French Tiger catheter over a 0.035-inch hydrophilic J-wire (Terumo Medical Corporation, Somerset, New Jersey) under fluoroscopy, which revealed 95% stenosis with a thrombus in the proximal left anterior descending (LAD) artery (Figure 1B). The other coronary arteries were normal.

Unfractionated heparin (70 U/kg) was administered during the intervention, guided by the activated clotting time (ACT) with a target value of 280-350 seconds. A 6 French Judkins Right 3.5 Guiding Catheter (Boston Scientific) was advanced over a 0.035-inch hydrophilic J-wire (Terumo Medical Corporation, Somerset, New Jersey) under fluoroscopy, and the LAD artery was engaged. The lesion was crossed with a TERUMO Run-through NS Extra Floppy 0.014-inch (0.36 mm) 180 cm guidewire. Lesion pre-dilatation was performed with a 2×15 mm semi-compliant balloon (Maverick, Boston Scientific), followed by stenting with a 3×23 mm Xience Expedition drug-eluting stent. TIMI (thrombolysis in myocardial infarction) 3 flow was achieved, and the procedure was completed without any complications (Figure 1C). During the procedure, the patient received a 25 μg/kg tirofiban bolus, followed by a maintenance dose of 0.15 μg/kg/min IV infusion.

Five hours after the procedure, the patient complained of sharp, pleuritic right-sided chest pain that increased with deep inspiration. On examination, he was tachypneic, tachycardic, and in respiratory distress. Respiratory examination revealed decreased chest excursion, decreased vocal fremitus, and reduced air entry on the right side. Percussion revealed stony dullness over the middle and lower parts of the thorax.

The chest radiograph revealed opacification of the right thoracic cavity (Figure 1D). 2D transthoracic echocardiography was apparently normal. A diagnostic pleural aspiration revealed bloody fluid with a hematocrit of 42. A complete blood count revealed a significant fall in hemoglobin from 15mg/dL to 8mg/dL with a normal platelet count. Contrast-enhanced computed tomography of the chest revealed a massive collection in the right pleural cavity with an attenuation of 35-70 HU and an underlying collapsed right lung, suggestive of a massive right hemothorax (Figure 1E).

We suspected iatrogenic injury to branches of the right subclavian artery or right internal mammary artery. We also ruled out any underlying chest pathology by doing a contrast-enhanced computed tomography of the chest. Tirofiban infusion was stopped immediately. An intercostal drainage (ICD) tube was inserted on the right side at the level of the fifth intercostal space, and 1000 mL of blood was collected in the drain leading to an immediate relief of his symptoms (Figure 1F). Diagnostic angiography through right transradial access of the right subclavian artery (Video 1), right internal mammary artery (Video 2), and a digital subtraction angiography of the right subclavian artery (Video 3) were performed, but no active bleeders were found.

**Video 1:** Angiogram of the right subclavian artery in the anteroposterior view, showing no oozing from any of the side branches and no breach in the intima of the vessels.

**Video 2:** Angiogram of the right internal mammary artery in the anteroposterior view, showing no evidence of a breach in the intima or oozing from the side branches.

**Video 3:** Digital subtraction angiogram of the right subclavian artery, showing the absence of any small vessel perforation or evidence of oozing.

The patient was then managed conservatively with the ICD in situ and transfusion of 2 units of packed red blood cells. A total of 800 mL of blood was collected in the ICD drain over a period of five days. The ICD was removed on day 5 as there was no output over the last 48 hours. The patient was discharged on guideline-recommended dual antiplatelet therapy and statin.

The ICD was removed on day 5 as there was no output for 48 hours. The patient was discharged on guideline-recommended dual antiplatelet therapy and statin. At the four-week follow-up, he was doing well, in NYHA Class I, and was able to perform all his routine activities without any complaints.

## Discussion

We report a rare case of right-sided massive hemothorax as a complication of angioplasty performed via the right radial artery approach. Although no active bleeder was found in this patient, bleeding complications can occur anywhere from the access site to the branches of the aorta along the route of the vessel. Our case is unique because all previous reports of similar cases revealed iatrogenic injury to large vessels, in contrast to our case, which revealed no vascular cause of bleeding. A plausible explanation for this could be an iatrogenic injury to one of the minor branches, which may have spontaneously thrombosed or gone into spasm. Another explanation could be that the bleeding resulted from capillary leakage under the effect of anticoagulation, due to the compounding effect of GpIIb/IIIa inhibitors and clexane given during the coronary angioplasty.

Variable predictors of major bleeding in a patient undergoing percutaneous coronary intervention (PCI) include the use of IABP, the administration of GP IIb/IIIa inhibitors, older age, female sex, chronic renal insufficiency, and the administration of low-molecular-weight heparin within 48 hours pre-procedure. Hydrophilic guidewires can also possibly cause perforation or hematoma in any branch of the passing artery [1,2].

Bleeding complications associated with the radial approach are significantly lesser compared to the femoral artery approach. Access site bleeding and mortality in high-risk PCI are known to be lower with the radial approach compared to the femoral approach. Despite a lower incidence of bleeding complications, radial artery access may be associated with life-threatening complications. Some possible severe complications include subclavian artery entrapment and perforation of the cephalo-brachial, thyroid, carotid, or internal mammary arteries. Wang et al. reported a case of massive thoracic hematoma following transradial coronary angiography [3]. Hours after the procedure, the patient complained of paroxysmal pain on the right side of the chest. Diagnostic angiography revealed a rupture of a branch of the right internal mammary artery. Endovascular occlusion therapy was performed successfully.

Hydrophilic wires may inadvertently enter even the smallest arterial branches with little resistance, leading to dissection, perforation, and hemorrhage. Angled wires have a higher tendency to enter small side branches compared with standard J-tip wires [4]. Choi et al. reported a case of a life-threatening mediastinal hematoma caused by a hydrophilic wire. During PCI, the patient rapidly developed voice changes, dyspnea, and lip cyanosis. Brachiocephalic angiography revealed a ruptured terminal branch of the inferior thyroid artery. Interventional occlusion and mediastinal hematoma aspiration were performed, and the patient eventually recovered [5]. Luo et al. observed that most hematomas in the thorax were related to hydrophilic guidewires during transradial cardiac catheterization [6].

Diffuse alveolar hemorrhage is a rare, life-threatening, and underdiagnosed complication of treatment with GP IIb/IIIa inhibitors. However, it is still unclear whether this bleeding complication is caused by tirofiban alone or by the combined effect of anticoagulation with aspirin, heparin, and clopidogrel.

Early detection of the complication is crucial to prevent a fatal outcome. Prompt bedside imaging and laboratory tests help in managing potential complications and facilitate timely intervention for the perforated vessel, thus improving patient prognosis. Table 1 summarizes previous similar case reports in which patients presented with hematomas secondary to coronary radial interventional procedures [7-12].

**Table 1:** Summary of hemothorax cases published following transradial coronary procedures. PCI, percutaneous coronary intervention

Author	Procedure	Complication	Course	Outcome	Management
Wang et al. [3]	Coronary angiography	Right internal mammary artery perforation leading to giant thoracic hematoma	Chest pain post-procedure. Imaging showed a 13.8× 6.7 cm thoracic hematoma.	Hemothorax drained; patient underwent valve replacements.	Endovascular coil embolization of right internal mammary artery branch; thoracic drainage.
Tatli et al. [7]	Coronary angiography	Right internal mammary artery perforation causing breast hematoma	Chest pain hours post-procedure. Large hematoma observed over the right breast.	Hematoma size reduced within hours post-stent implantation.	Stent graft implantation to seal perforation.
Park et al. [8]	PCI	Inferior thyroid artery perforation leading to mediastinal hematoma and hemothorax	Cough, dyspnea, and stridor during procedure. Imaging showed mediastinal hematoma and hemothorax.	Successful hemostasis; Patient remained healthy at 14-month follow-up.	Endovascular embolization with micro-coil, gelatin sponge, and glue; mediastinal hemorrhage drained via mediastinoscopy.
Bhatia et al. [9]	Transradial catheterization	Axillary artery perforation leading to pectoral hematoma	Right pectoral swelling 30 minutes post-procedure.	Hematoma resolved by day 7; Patient remained stable.	Compression bandage, conservative management.
Wu et al. [10]	Coronary angiography	Multiple-site bleeding at pleural adhesions causing massive hemothorax	Chest pain and dyspnea post-PCI. Imaging revealed hemothorax.	Hemothorax resolved after surgical intervention; patient recovered without complications.	Surgical exploration and hemostasis at bleeding sites.
Cho et al. [11]	Cerebral angiography	Mediastinal and thoracic hematoma	Chest discomfort and dyspnea during procedure. Imaging showed mediastinal and thoracic hematoma.	Hematoma resolved with conservative management.	Conservative management with close monitoring.
Lemos et al. [12]	Coronary angiography	Subclavian artery pseudoaneurysm leading to hemothorax	Chest pain and swelling post-procedure; imaging revealed pseudoaneurysm and hemothorax.	Pseudoaneurysm treated successfully; patient recovered.	Endovascular stent graft placement.

## Conclusions

Hemothorax is a rare but unforgiving complication of the transradial approach for coronary intervention, which may prove fatal if not detected early. The use of ordinary Teflon wire, as compared to hydrophilic wires, helps decrease iatrogenic injury to small vessels. One must always insert the wire under fluoroscopy and should avoid pushing the wire whenever resistance is encountered. Additionally, ACT monitoring is mandatory, especially in patients where longer procedures are anticipated or when tirofiban is used. 

Early intervention and a prompt search for the culprit injured vessels help control the bleeding. Conservative management with an ICD for hematoma to relieve the symptoms, along with selective angiography to rule out injury to the major vessels, seems a reasonable approach in such cases. In most instances, a vessel injury is found as the etiology of the hemothorax and should be addressed by percutaneous intervention.
